# Inclusion of Limited Amounts of Extruded Legumes Plus Cereal Mixes in Normocaloric or Obesogenic Diets for Rats: Effects on Lipid Profile

**DOI:** 10.3390/foods9060704

**Published:** 2020-06-01

**Authors:** Luis A. Rubio, Isabel Aranda-Olmedo, Mercedes Martín-Pedrosa

**Affiliations:** 1 Department of Physiology and Biochemistry of Animal Nutrition, Estación Experimental del Zaidín (EEZ, CSIC), Profesor Albareda, 1, 18008 Granada, Spain; isabel.aranda@eez.csic.es; 2 SGIT-INIA, Tecnología de los Alimentos, Ctra. de la Coruña Km 7, 28040 Madrid, Spain; mmartin@inia.es

**Keywords:** extrusion, legume, lipid metabolism, obesity, rat

## Abstract

Overweight and obesity are regarded as world epidemics and are major risk factors for a number of chronic diseases, including diabetes, cardiovascular diseases, and cancer. Two new highly palatable extruded mixes based on rice and pea (*Pisum sativum*) or kidney bean (*Phaseolus vulgaris*) meals were incorporated into normocaloric or obesogenic diets for rats at a low inclusion level (25%). Our purpose was to evaluate the effects of dietary incorporation of this new food ingredient on lipid profile. Organs (heart, liver, kidneys, spleen, stomach, small intestine, colon, cecum) and visceral fat relative weights were different (*p* < 0.01) from controls for animals fed the obesogenic diets and in rats fed extruded diets with respect to controls. Faecal excretion of bile acids was higher (*p* < 0.01) for rats fed extruded mixes compared with controls. The inclusion of extruded mixes replacing part of the casein in the control diet lowered liver cholesterol and triglycerides (*p* < 0.001) and plasma low-density lipoprotein (LDL; *p* < 0.01) values, although plasma high-density lipoprotein (HDL) was unaltered. Both the inclusion of extruded mixes and the use of obesogenic diets resulted in significantly (*p* < 0.001) different long chain fatty acid (LCFA) profiles in liver and visceral fat. Incorporating extruded legume plus cereal mixes beneficially influenced lipid metabolism, and would therefore deserve closer attention in human intervention studies, particularly with adolescents. To our knowledge, this is the first report on the nutritional and physiological effects of extruded legume plus cereal mixes.

## 1. Introduction

Overweight and obesity are defined as abnormal or excessive fat accumulation that presents a risk to health. According to the WHO [1], overweight and obesity are regarded as world epidemics and are major risk factors for a number of chronic diseases, including diabetes, cardiovascular diseases, and cancer. Once considered a problem only in high-income countries, overweight and obesity are now dramatically on the rise in low- and middle-income countries, particularly in urban settings. Even more worrying is the fact that the prevalence is steadily increasing in children and adolescents, both in developed and developing countries [2]. In 2011, more than 40 million children under five years of age suffered from or were overweight [3].

Obesity is associated with metabolic derangements in multiple tissues, which contribute to the progression of insulin resistance and metabolic syndrome. This has been defined as a cluster that requires at least three of five factors: increased waist circumference, hypertriglyceridemia, low high-density lipoprotein (HDL) cholesterol, hypertension, and a fasting glucose of 110 mg/dL or higher [4]. Obesity is also associated with the Western diet [5]. Eating patterns in Western industrialised countries are characterised by high energy consumption and chronic overconsumption of saturated fat, cholesterol, sugar, and salt, which is also related to the development of other pathologies such as diabetes, cardiovascular and degenerative disorders, and cancer [6]. In this context, it has been proposed that weight loss could be achieved with controlled energy diets and a high content of cereals and legumes [7]. Legumes have long been an important component of the human diet because of their content in protein, carbohydrates (mainly in the form of starch), and many other nutrients such as proteins, and fibre [8], but in addition, the consumption of legumes can have therapeutic effects, being an important element of the Mediterranean diet pattern [9]. Additionally, legume carbohydrates, known as “slow-release carbohydrates”, when added to the diet are an effective tool in the treatment of obesity, diabetes, and hyperlipidemia [10]. The substitution of energy-rich foods for pulses has been reported to have beneficial effects on the prevention and treatment of obesity and related disorders such as cardiovascular diseases, type 2 diabetes, and metabolic syndrome [10,11,12].

Recommendations to increase the fibre intake in the child/adolescent population emphasise the greater consumption of fruits, vegetables, pulses, and whole grains. Given the difficulties of changing the dietary habits of the population, one way to optimise fibre intake would be through the consumption of other types of fibre-supplemented foods [13]. Alternative foods with high palatability would be particularly useful in this context. Accordingly, the present work aimed to add some more information to the study of the effects that the consumption of legumes and cereals may have on health through the evaluation of new products derived from these foodstuffs that have been adapted to the present consumer demands. These demands in Western societies no longer respond so much to the need to satisfy hunger or the need of energy, but to the “hunger of health” or absence of disease.

In this context, new highly palatable [14] extruded mixes of legumes plus cereals similar to a snack food have been produced [15]. Two of these extruded mixes have been incorporated in the current work into normocaloric or obesogenic diets for rats to evaluate the effects on lipid profile and intestinal microbiota composition [16]. Obesogenic diets are those that promote obesity by presenting high caloric contents mainly from a high proportion of carbohydrates, usually simple sugars and/or fats [17]. As the intake of this new snack type food in practical conditions would be necessarily limited, we included it in the diet at a low inclusion level (25%) to provide no more than 20% of the total dietary protein. Our purpose was to evaluate the effects of dietary incorporation of this new food ingredient on lipid profile.

## 2. Materials and Methods

All management and experimental procedures carried out in this rat trial were in strict accordance with current European regulations (86/609 E.E.C.) regarding laboratory animals. The Bioethics Committee for Animal Experimentation at our institution (EEZ-CSIC) approved the study protocol.

### 2.1. Preparation of the Extruded Mixes and Diets

Two extruded legumes plus cereal mixes were produced by using a Clextral Evolum25 twin-screw extruder (Clextral, Riez 42702 Firminy Cedex, France). These were rice (*Oryza sativa*) + pea (*Pisum sativum*, cv Cartouche), or kidney bean (*Phaseolus vulgaris*, cv Almonga) + carob tree (*Ceratonia siliqua*) fruits. Extruded mixes (PEM and KEM) composition was rice meal/pea or kidney bean meal/carob fruits meal, respectively (50/40/10, w:w:w) and contained no casein. All procedures were carried out as described previously [15]. The chemical composition of casein, and extruded mixes are shown in Table 1.

The diets (Table 2) were based on casein (CAS) or casein plus pea or kidney bean extruded mixes (PEM and KEM) and were formulated to contain the same amount of digestible energy and protein. In order not to be too far from practical conditions in human nutrition, PEM and KEM addition was limited to a low inclusion level (25%) to provide no more than 20% (two servings) of the total recommended daily protein intake. Another relevant point on formulation in the present work was that diets contained no added cholesterol. Appropriate amounts of synthetic amino acids were added to the extruded mix-based diets, taking into account their amino acid composition (Table 1) to equalise them to control (casein) values. The diets were supplemented with vitamins and minerals to target requirements [18]. Obesogenic diets (CAS-OB, PEM-OB, and KEM-OB) were the same diets described above (CAS, PEM, and KEM) with added amounts (5.48 g/day) of IDEAL commercial condensed milk (composition in Table 2).

### 2.2. Biological Assays

A total of 72 male weaned Wistar rats (Charles River Laboratories), matched by weight (80.0 ± 2.7 g, mean ± SE) and with 4 weeks of age, were individually housed in metabolism cages throughout the experiment. Rats were fed a control casein diet between weaning and the start of the experiment. Animals were then randomly distributed into six groups (*n* = 12), and each group was assigned to one of the dietary treatments (see above). The animals were individually housed in metabolic cages in an environmentally controlled room under standard conditions (temperature: 20–22 °C with a 12 h light–dark cycle and 55–70% humidity). The rats had free access to their diets and tap water, and were fed the different diets for 21 days. Feed offered was calculated to be 95% of ad libitum intake for this age and species, and was progressively increased according to rats’ age. Rats ate all feed offered and there were no leftovers. Faeces were collected daily and stored separately as a 1-week pool for each animal. The faeces were weighed, lyophilised, powdered, and then homogenised. On day 21, after an overnight fast, they were refed at defined time intervals with 11 g of diet and euthanized 60 min after refeeding so that all rats were in the same feeding situation. Animals were anaesthetised with sodium pentobarbital (5 mg/100 g of body weight) (Abbott Laboratories, Granada, Spain) and terminal exsanguination was performed by cannulation of the carotid artery. Organs and visceral fat (defined as the sum of the mesenteric, epididymal, and perirenal fat depots) were removed and weighed, and intestinal contents were immediately frozen at −20 °C and then freeze-dried until analysis.

### 2.3. Chemical and Biochemical Analysis

All chemicals were obtained from Sigma-Aldrich Química S.L. (Madrid, Spain), unless otherwise stated. Proximate analysis (Table 1) of casein and extruded mixes was carried out following AOAC (Association of Official Agricultural Chemists) procedures as in Arribas et al. [15], and amino acids were determined by the Pico Tag method in an HPLC analyser (Waters, Milford, MA, USA) as in Rubio et al. [19]. α-Galactosides were determined by HPLC equipped with a refractive index detector according to Pedrosa et al. [20].

Plasma cholesterol and triglycerides analysis was performed in a BS-200 Biochemical Analyzer (Shenzhen Mindray Bio-medical Electronics Co., Ltd., Mindray Medical España S.L., Madrid, Spain) with chemicals from Spinreact (Spinreact, S.A.U., Gerona, Spain). Liver cholesterol and triglycerides were determined by using colorimetric/fluorometric methods from Abcam kits (ab65359 and ab65336 for cholesterol and triglycerides, respectively).

Long chain fatty acids (LCFA) in liver and visceral fat were determined as in Palmquist and Jenkins [21]. Briefly, samples (0.3 g) were taken in test tubes with Teflon screw caps and kept at 4 °C. Internal standard (C23:0, Larodan’s methyl tricosanoate ref. 20-2300; 4 mg in 2.0 mL n-heptane) were added, followed by 1.8 mL of 10% methanolic HCl. The tubes were tightly capped, vortexed, heated in a water bath at 90 °C for 2 h, cooled, and then 0.6 mL of heptane and 6 mL of 6% K_2_CO_3_ were added. Tubes were then vortexed and centrifuged at 4 °C for 5 min at 500× *g*. The top layer (organic solvent) was transferred to a tube with a screw Teflon cap containing 0.6 g of sodium sulphate. After vortexing again and centrifuging at 4 °C for 5 min at 500× *g*, the solvent layer was transferred to 2 mL GLC (gas liquid chromatography) sample vials, which were analysed by GLC.

Bile acids in faeces were determined as in Hagio et al. [22]. Briefly, faeces were freeze-dried, ground, extracted in ethanol, sonicated, heated twice in a water bath, and centrifuged. This procedure was repeated twice, and the pooled extracts were evaporated and dissolved in methanol. The methanol extracts were purified and bile acids were analysed by liquid chromatography (LC) using an Acquity UPLC system (Waters) with a gradient elution from a BEH C18 column (1.7 mm, 100 mm × 2.0 mm ID; Waters).

### 2.4. Statistical Analysis

Statistical significance of data was tested by two-way (+/− extruded mix addition, +/− obesogenic diet) analysis of variance (ANOVA) with Tukey’s post-hoc test with statistical significance set at *p* < 0.05. Evaluation of the relationship between the different variables of interest was carried out by computing the relevant correlation coefficient (Pearson’s linear correlation). For qPCR microbial counts, discriminant analysis (DA) was used to check if the groups to which bile acids or LCFA observations belonged to were distinct, and principal component analysis (PCA) was used to study the relationships between bacterial groups [23].

## 3. Results

### 3.1. Growth and Feed Intake

**Figure 1:**
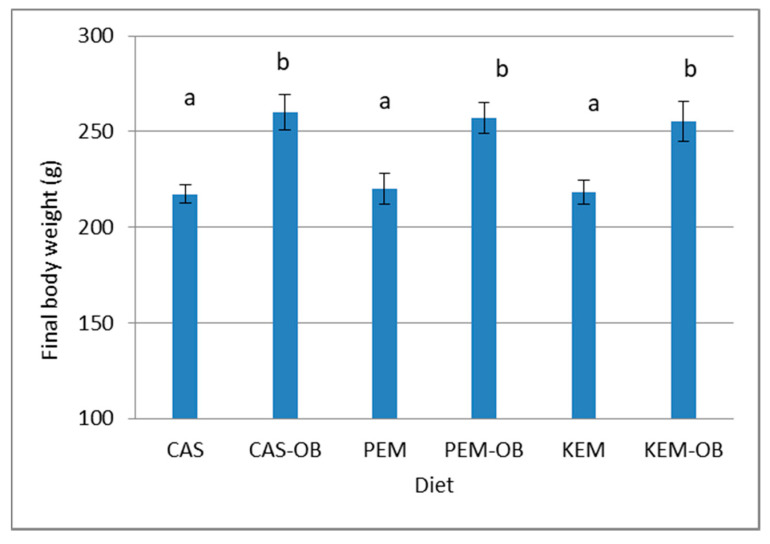
Final body weight of rats fed casein or casein + extruded mix diets either obesogenic or not. CAS, casein diet; KEM, kidney bean (*Phaseolus vulgaris*) extruded mix diet; PEM, pea (*Pisum sativum*) extruded mix diet. Diets CAS-OB, KEM-OB, and PEM-OB were the same diets described above with added 5.48 g/day condensed milk. Values are means (*n* = 12) with their standard error of the mean (SEM) in bars. ^ab^ Means with different superscript differ (*p <* 0.01).

Food consumption (Table 3) and final body weight (Figure 1) was the same among non-obesogenic groups and among obesogenic groups, and different (*p* < 0.01) between non-obesogenic and obesogenic groups.

### 3.2. Organs and Visceral Fat Relative Weights

Carcass, organs (heart, liver, kidneys, spleen, stomach, small intestine, cecum, and colon), and visceral fat relative weights (g/100 g fresh weight) of rats fed the different diets are recorded in Table 4. Carcass weight was higher (*p* < 0.01) than controls for rats fed extruded diets, and liver weight was higher (*p* < 0.01) for rats fed obesogenic diets compared with non-obesogenic diets. Visceral fat relative weights were higher (*p* < 0.01) for the animals fed the obesogenic diets in comparison with non-obesogenic diets, and in rats fed extruded diets respect to the control (CAS or CAS-OB) diets. A significant interaction (*p* = 0.034) was found between extrusion and obesogenic effects for visceral fat. Rats fed extruded mixes had lower (*p* < 0.01) small intestine, colon, and cecum relative weights compared with controls.

### 3.3. Bile Acids in Feces

**Figure 2:**
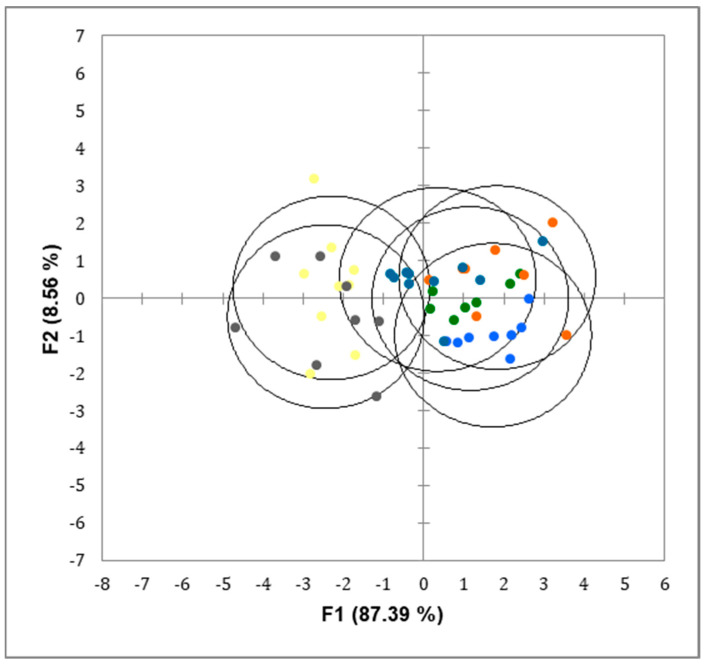
Discriminant analysis of the expression levels of bile acid concentration (μM/g) in the faeces of rats fed CAS (yellow); CAS-OB (grey); KEM (orange); KEM-OB (dark blue); PEM (light blue); PEM-OB (green). For details on the diets, see the Materials and Methods section and Table 2. Values for rats fed diets based on casein (CAS and CAS-OB) were different (*p <* 0.05) from those fed diets based on extruded mixes, although not different to each other.

Fecal excretion of cholic, chenodeoxycholic and ursodeoxycholic acids was lower (*p* < 0.05) and deoxycholic acid and total bile acids higher (*p* < 0.01) for rats fed extruded mixes compared with CAS and CAS-OB controls (Table 5). Obesogenic diets did not induce any significant change in bile acids fecal excretion. However, there was a significant (*p* < 0.05) interaction between extrusion and obesogenic effects for total bile acids. As shown in Figure 2, values for rats fed diets based in casein (CAS and CAS-OB) were different (*p* < 0.05) from those fed diets based in extruded mixes.

### 3.4. Liver and Plasma Cholesterol and Triglycerides

Results on liver and plasma cholesterol and triglycerides are reported in Table 6. The inclusion of the extruded mix replacing part of the casein in the control diet (CAS) lowered liver cholesterol and triglycerides (*p* < 0.001) and plasma low-density lipoprotein (LDL; *p* < 0.01) values, although plasma HDL was unaltered. The obesogenic diets induced lower (*p* < 0.017) liver cholesterol values with respect to non-obesogenic diets only in the diet with the extruded legumes plus cereal mix. The obesogenic diets increased (*p* < 0.05) plasma triglyceride values with respect to the non-obesogenic diets.

### 3.5. Long Chain Fatty Acid Composition of Liver and Visceral Fat

As shown in Table 7, the livers of rats fed diets containing extruded mixes (KEM or PEM) had higher proportions of C10, C15:1, C16, C17:1, C18, C18:1 11c, C18:2, C20, and C20:3, and lower proportions of C20:4 and C22:6 than CAS controls. Obesogenic diets increased the proportions of C16:1, C18, C18:1 9c, and C18:1 11c, and lowered those of C18:2 n6 9c12c, C20, C18:3 n6, C20:2 n6, C24, and C24:1. The inclusion of the extruded mixes induced an increase in the proportions of monounsaturated fatty acid (MUFA) in the liver, while the obesogenic diets caused increases of both MUFA and polyunsaturated fatty acid (PUFA) in the liver. No significant interactions between extruded mix and obesogenic diets were found in LCFA liver composition.

The visceral fat (Table 8) of rats fed the extruded mixes had higher proportions of C16, C16:1, and C18:1 9c, and lower proportions of C18:2 n6. The obesogenic diets induced increases of C10, C12, C14, C16, C16:1, C17, C18:1 9c, and C18:1 11c, and decreases of C18:2 n6, C20:2 n6, and C20:4. Extruded mixes and obesogenic diets induced an increase in the proportions of SFA and MUFA, and a decrease of PUFA proportions in the visceral fat.

**Figure 3:**
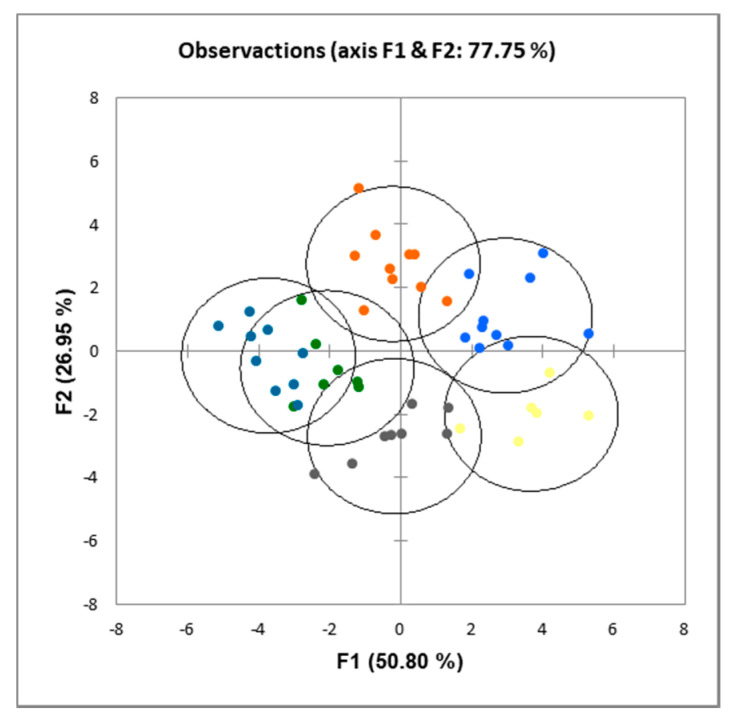
Discriminant analysis of liver fatty acid composition of rats fed CAS (yellow); CAS-OB (grey); KEM (orange); KEM-OB (dark blue); PEM (light blue); PEM-OB (green). For details on the diets, see the Materials and Methods section and Table 2. All groups were different (*p* < 0.001) from each other.

**Figure 4:**
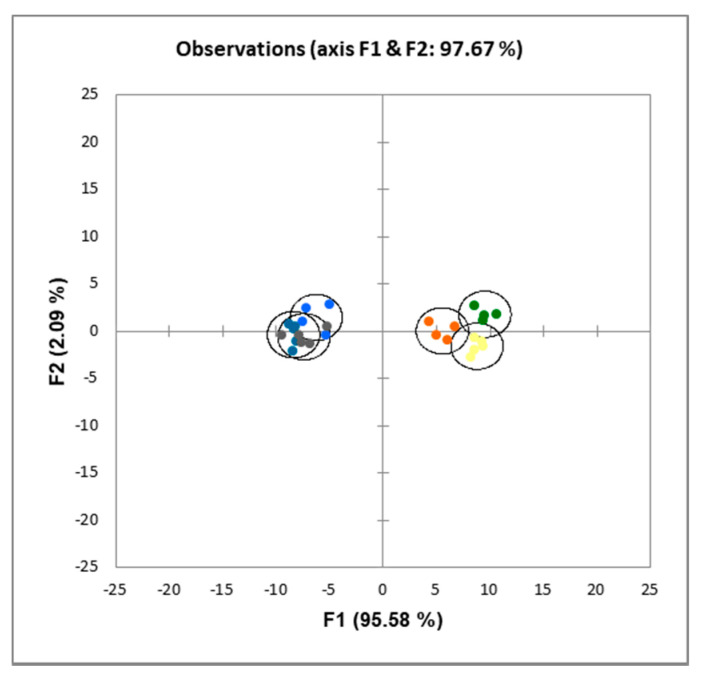
Discriminant analysis of visceral fat fatty acid composition of rats fed CAS (yellow); CAS-OB (grey); KEM (orange); KEM-OB (dark blue); PEM (green); PEM-OB (light blue). For details on the diets, see the Materials and Methods section and Table 2. Groups CAS-OB, PEM-OB, and KEM-OB were different (*p* < 0.01) from CAS, PEM, and KEM, but not different to each other.

Discriminant analysis (Figure 3 and Figure 4) of the data showed that both the inclusion of extruded mixes and the use of obesogenic diets resulted in significantly (*p* < 0.001) different LCFA profiles in the liver and in the visceral fat.

## 4. Discussion

According to the United States Department of Agriculture (USDA) food pattern recommendations, for a 2000 kcal diet, one serving (40 g) of extruded blends similar to those used here would provide (on average) 11% of daily protein intake, 0.1% of daily fat intake, and 25% of daily carbohydrate intake [15]. The inclusion of either of the extruded mixes of legumes plus cereals described above in casein-based diets for rats replacing no more than 20% of the protein had significant effects on lipid profile. As a preliminary indication, we must point out here that most of the discussion below will lie on the legume portion of the extruded mix for two main reasons: (i) legumes are usually mixed with starch of different sources, mainly corn or rice starch, in order to obtain puffed products, and (ii) very scant information is found in the literature on the nutritional effects of extruded rice in vivo.

Health professionals and agencies such as the USDA and Health Canada are promoting dietary changes including increased consumption of grain legumes (or pulses) such as peas, kidney beans, lentils, and chickpeas (www.pulsecanada.com). In the current work, pea and kidney bean seeds were used to produce the extruded composites, which were incorporated into non-obesogenic diets (PEM and KEM) formulated to be equivalent in energy and protein to a control diet (CAS). Condensed milk was added to the daily food intake to produce the obesogenic diets (CAS-OB, PEM-OB, KEM-OB) (Table 2). This ingredient has been previously utilised in well-characterised rat models of hyperphagia-induced obesity [24,25]. As expected, rats fed the obesogenic diets showed substantially higher intakes of food (36.73%), energy (36.50–36.95%), protein (16.40%), fat (51%), and carbohydrates (41.98–42.23%) (Table 3). This resulted in higher final body weights (Figure 1) and visceral fat relative weights (Table 4) with respect to non-obesogenic controls in rats fed the obesogenic diets, which is similar to previous results with this type of diets [25]. However, the incorporation of the extruded mixes at the relatively low levels used here (250 g/kg) had a significant effect on liver fat composition (Table 6) and on the amount and composition of visceral fat (Table 4 and Table 7). This is not in agreement with previous work, where lower fat deposition has usually been reported in rats fed obesogenic diets based on legume (particularly soy) protein [26], albeit with all or most of the control protein replaced by legume protein in most cases. As explained above, our aim in the current work was to evaluate the effects of incorporating the extruded mix in the diet to replace only a limited amount of the protein in the control diet (closer to practical consumption). The literature on the effects of limited amounts of legume seed meals, either heat treated or not, on growth or physiological parameters in rats is quite scarce. At this level of inclusion, legumes in general, and kidney beans or peas in particular, have not been reported to affect growth rate or lipid deposition, particularly after heat treatment and supplementation with limiting amino acids [27,28].

Legume-based diets have been associated with changes in organs relative weights in rats [29]. In the current study, rats fed extruded mixes had lower small intestine, colon, and cecum relative weights compared with controls (Table 4). This is contrary to previous works, where higher relative weights of the intestinal sections have usually been reported and ascribed to the presence of higher amounts of indigestible material (particularly dietary fibre) and/or an increased production of short chain fatty acids (SCFA), which have a stimulating effect on gut epithelial proliferation [30,31]. This discrepancy is likely due to the lower dietary inclusion levels in the current study, which resulted in smaller differences in dietary fibre (Table 2) as compared to other studies. However, in a previous work, we found lower cecum, rectum, and large intestine relative weights, as well as a tendency for small intestine relative weight, in rats fed diets based on isolated lupin (*Lupinus albus*) protein [32]. It has previously been reported that soy proteins reduce colon weight in mice in comparison to casein, an effect ascribed to reduced cell proliferation [33]. Since legumes form an important part of the “prudent” diet that may be associated with reduced colon cancer risk in humans [34], the effect of legume protein consumption on the reduction of large intestinal weight and the relationship between intestinal weight changes and intestinal cell proliferation/carcinogenesis is worthy of further investigation.

Liver and visceral fat relative weights were as expected higher for the animals fed the obesogenic diets in comparison with non-obesogenic diets. However, carcass and visceral fat weight were also higher than controls in rats fed the extruded diets (Table 4). This was unexpected since there are no indications on fat retention in animals fed diets containing extruded legumes. Three explanations may be adduced for higher energy absorption and thus higher lipid accumulation in extruded supplemented diets. Firstly, extruded mix-supplemented diets used herein contained between 47–57% higher amounts of fibre than controls (Table 2). According to McBurney and Sauer [35], hind gut fermentation of fibre would provide as much as 13 kJ/g fermentable dietary fibre coming from SCFA absorption, and 40–57 g non-starch polysaccharides (NSP)/100 g ingested was fermented in rats fed faba bean- or chick pea-based diets [36]. Thus, the diets containing the extruded mixes would have resulted in a greater energy utilisation of the diet due to the higher amounts of SCFA produced and absorbed [37,38,39]. Secondly, bile acid excretion was higher in rats fed diets containing extruded mixes (Table 5). Dietary lipids, phospholipids, and cholesterol are solubilised by way of the detergent capacity of bile acids [40], and thus higher intestinal bile acids concentrations would facilitate lipids absorption. Finally, proximate analysis of extruded materials does not lack specific complications [15], which makes it more difficult to accurately establish their protein, carbohydrate, and lipid concentrations, and consequently their energy content for dietary formulation.

Pulses have traditionally been consumed mostly after some kind of heat treatment for gastronomic reasons, but also to reduce the effect of a number of bioactive compounds that in some cases have deleterious effects [41,42]. Compared with the traditional processing methods, extrusion processing, which involves a combined thermo-mechanical treatment with moisture and temperature control, is a quicker and more consistent way to cause thermal/chemical breakdown of deleterious bioactive compounds and at the same time could alter the physical, chemical, and nutritional nature of nutrients in a desirable manner [43]. In fact, pea and kidney bean extrusion treatment has shown a positive effect on mineral, in vitro protein, and starch digestibilities and in vivo nutritional utilisation [44,45,46]. Even more, extrusion cooking improved the nutritional quality of pea seeds without reducing their hypocholesterolemic and triglyceride-lowering properties [28,30], which is in keeping with our current results (Table 6). However, those previous results were obtained with a more than double (570 g/kg) dietary level of inclusion than those used here. Another relevant point in this investigation was that we used no manipulation of cholesterol metabolism by feeding dietary cholesterol, which is in contrast to what has been previously reported in the literature, where the effects of bean feeding were generally assessed on diet-induced hypercholesterolemia. Additionally, in working with low inclusion levels (100 g/kg) and no cholesterol addition, McPherson [47] found a lowering effect of cooked kidney bean on blood cholesterol. This indicated that legume intake is likely to be a very effective means for keeping low circulating cholesterol values, and that it is able to disturb endogenous cholesterol metabolism, which was also confirmed in the current work by the lowering of liver cholesterol values (Table 5). The mechanisms related with this effect have been linked to the combined effects of a number of bioactive substances and to the protein and fibre [48] or starch [49] fractions of the seed. Legume seed meals and protein isolates are known to modulate plasma amino acid concentrations [29,30,50,51] and induce other physiological effects including hypocholesterolaemia mediated by hormonal (insulin, glucagon) shifts [52,53]. Other reported mechanisms include up-regulation of hepatic mRNA levels of Sterol Regulatory Element-Binding Protein 2 (SREBP-2), a major transcriptional regulator of intracellular cholesterol levels, and Cytochrome P450 7A1 (CYP7A1), the rate-limiting enzyme in bile acid biosynthesis [54], or downregulation of fatty acids synthesis [55,56]. The hypocholesterolemic effect has also been linked to differences in the dietary Lys/Arg and Met/Gly ratios [51,57,58]. In the current work, these ratios for the different diets (Lys/Arg: CAS 2.62, KEM 1.99, PEM 1.92; Met/Gly: CAS 2.35, KEM 0.85, PEM 0.81) were quite different due to differences in amino acid contents in casein and extruded mixes (Table 1). However, these ratios have been found to be more effective in regulating triglyceride than cholesterol values [50,57]. In a laboratory trial using non-overweight and diet-induced obese rats, short-term dietary intake of kidney beans reduced the total plasma cholesterol and low-density lipoprotein (LDL) cholesterol, without affecting high density lipoprotein (HDL) cholesterol or plasma total triglycerides levels [59], which is in line with the current results (Table 6). This would be also in line with the increased faecal bile acids here reported (Table 5), since increased bile acid excretion has also been claimed as a mechanism to explain the cholesterol lowering effect of legume protein-based diets through removal of blood cholesterol via the LDL receptor [48] or bile acids binding within the intestine [60]. Finally, bile acid metabolism is known to be modulated by gut microbiota, affecting the biotransformation, reabsorption, and excretion of bile acids by catalysing a range of biochemical reactions [61]. Nakatani et al. [62] have recently found an elevated cecal and faecal bile acid pool in mice that had consumed mung bean protein, and those changes were linked to dramatic changes in the gut microbiome, such as such as changes in the Firmicutes/Bacteroidetes proportions (see Rubio et al. [16]).

The dietary inclusion of extruded kidney bean or pea plus rice mixes resulted in significant changes in the proportions of LCFA both in liver and visceral fat. Thus, the inclusion of the extruded mixes induced an increase in the proportions of MUFA in the liver, and of SFA and MUFA in the visceral fat, while the obesogenic diets gave place to an increase in MUFA and a decrease in PUFA in the liver and increases of SFA, MUFA, and a decrease in PUFA in the visceral fat (Table 7 and Table 8). Moreover, dietary inclusion of extruded legumes plus cereal mixes gave place to significantly different fatty acid profiles in both liver and visceral fat (Figure 3 and Figure 4) when compared with the casein control diet. Very scant information can be found in the literature thus far on the effects of legume feeding on long chain fatty acid metabolism. In the current work, it is noteworthy that the fat composition of the diets was almost identical, as all of them contained sunflower in the same proportions (Table 2), and the amounts of fat in extruded mixes was lower than 0.2% (Table 1). Therefore, the differences in protein and/or fibre composition of KEM and PEM with respect to CAS are likely to be the ultimate reason for the differences found in this work. In 1992, Ogawa et al. [63] reported that the fatty acid composition of liver phospholipids was influenced by the type of soybean protein used. Quite interestingly, these authors found an increase respect to CAS in C18:2 n-6, and a decrease in C22:5 n-6 in serum and adipose tissue, which is in keeping with the results reported here in liver fat (Table 7) but not in visceral fat (Table 8), albeit with a much lower amount of legume protein. Legume feeding to cows has been reported to induce changes even in milk fatty acids composition [64], most likely due to changes in ruminal fermentation. Finally, obesogenic diets also gave place to increases in SFA and MUFA and decreases in PUFA, presumably due to the much higher amounts of SFA and MUFA in milk fat [65], which is in line with our current results.

## 5. Conclusions

The substitution of energy-rich foods for pulses has been reported to have beneficial effects on the prevention and treatment of obesity and related disorders such as cardiovascular diseases, type 2 diabetes, and metabolic syndrome. Alternative foods with high palatability such as extruded mixes would be particularly useful particularly in the context of child/adolescent population. The dietary use of limited amounts (closer to practical conditions) of an extruded legumes plus cereal mix did modulate carcass, visceral fat, small intestine, colon, and cecum relative weights in rats. Faecal excretion of bile acids was also affected in rats fed extruded mixes, while obesogenic diets did not induce any significant change in bile acids faecal excretion. The inclusion of extruded mixes lowered liver cholesterol and triglycerides and plasma LDL values, and resulted in different LCFA profiles in liver and visceral fat. To our knowledge, this is the first report on the nutritional and physiological effects of extruded legume plus cereal mixes. The results reported here reinforce the contention on the health benefits of incorporating these kinds of food ingredients into the human diet, and would therefore deserve closer attention in human intervention studies, particularly with adolescents.

## Figures and Tables

**Table 1:** Analysed composition (g/kg) of casein and the legume + cereal extruded mixes.

	Casein	Extruded Pea Mix ^1^	Extruded Kidney Bean Mix
Dry matter	920.0	944.4	947.6
Protein	813.3	128.2	132.3
Ether extract	ND ^2^	1.2	1.4
Ash	ND	34.6	38.4
Crude fibre	ND	32.1	24.6
Dietary fibre	ND	94.1	114.5
α-galactosides	ND	32.8	25.8
Total carbohydrates ^3^	ND	653.5	635.2
Crude energy (Kcal/g)	3.940	3.600	3.620
Amino acids			
Aspartate	58.9	15.1	15.8
Glutamate	184.1	25.4	26.2
Serine	49.1	6.0	7.3
Histidine	16.5	3.3	4.1
Glycine	27.5	6.1	6.1
Threonine	39.6	4.4	5.0
Arginine	37.4	12.5	11.4
Alanine	32.6	6.8	7.0
Tyrosine	49.0	5.5	5.7
Cystine	3.4	1.5	1.4
Valine	53.3	7.0	7.5
Methionine	24.5	1.8	2.5
Phenylalanine	46.9	7.2	8.1
Isoleucine	40.9	5.9	6.3
Leucine	74.9	10.9	11.9
Lysine	97.9	7.2	7.5
Proline	82.8	6.6	6.1

^1^ Pea (*Pisum sativum*) extruded mix (rice/pea/carob tree bean, 50/40/10); kidney bean (*Phaseolus vulgaris*) extruded mix (rice/kidney bean/carob tree bean, 50/40/10). ^2^ ND, not determined. ^3^ Total solids excluding protein, fat, ash, dietary fibre, and α-galactosides.

**Table 2:** Composition (g/kg) of the diets.

	CAS ^1^	PEM	KEM
Extruded mix	-	250	250
Casein	209	160	160
Maize starch	261.28	209.58	209.58
Potato starch	150	-	-
Cellulose	50	50	50
Sunflower oil	70	70	70
Sucrose	150	150	150
DL-Methionine	3	3	3
Tryptophane	-	0.70	0.70
Coline	1.60	1.60	1.60
Cystine-HCl	1.80	1.80	1.80
Vitamins + minerals	45	45	45
Ca diphosphate	55	55	55
Citric acid	0.12	0.12	0.12
Iron sulfate heptahydrate	0.20	0.20	0.20
Cr_2_O_3_	3	3	3
Calculated composition			
Energy (kcal/g)	3.23	3.22	3.21
Protein	170	170	170
Fat	70	70	70
Carbohydrates	486.3	531.3	525.8
Dietary fibre	50.0	73.5	78.6

^1^ CAS, casein diet; KEM, kidney bean (*Phaseolus vulgaris*) extruded mix diet; PEM, pea (*Pisum sativum*) extruded mix diet.

**Table 3:** Total daily feed intake (g/day) of rats fed casein or casein plus extruded mix diets either obesogenic or not for 21 days.

	CAS	CAS-OB	PEM/KEM	PEM-OB/KEM-OB
Intake (g)	15.71	21.48	15.71	21.48
Energy (kcal)	48.30	66.15	48.00	65.52
Protein (g)	2.54	2.95	2.54	2.95
Fat (g)	1.05	1.51	1.05	1.51
Carbohydrates (g)	7.27	10.34	7.86/7.94	11.16/10.97

**Table 4:** Organs relative weight (g/100 g fresh body weight) of rats fed casein or casein plus extruded mix diets either obesogenic or not.

	Carcass	Heart	Liver	Kidneys	Visceral Fat	Spleen	Stomach	Small Intestine	Colon	Cecum
CAS ^1^	76.474	0.310	4.022	0.950	1.398	0.350	0.596	3.775	0.483	1.186
CAS-OB	77.685	0.316	4.226	0.891	2.127	0.350	0.560	3.544	0.405	1.175
PEM	79.701	0.304	4.018	0.949	1.606	0.376	0.605	3.322	0.406	0.490
PEM-OB	79.535	0.306	4.413	0.964	2.922	0.382	0.556	3.272	0.411	0.455
KEM	79.884	0.318	3.982	0.925	1.601	0.386	0.600	3.210	0.374	0.490
KEM-OB	78.924	0.328	4.193	0.943	2.615	0.409	0.620	3.268	0.386	0.492
Pooled SD	1.744	0.024	0.377	0.071	0.500	0.065	0.073	0.278	0.070	0.214
*p-Values* ^2^										
E	<0.001	0.831	0.484	0.067	<0.001	0.238	0.258	<0.001	0.001	<0.001
O	0.765	0.155	0.003	0.649	<0.001	0.793	0.110	0.122	0.120	0.378
E × O	0.177	0.889	0.376	0.041	0.034	0.529	0.767	0.272	0.064	0.983

Values are means (*n* = 12). ^1^ CAS, casein diet; KEM, kidney bean (*Phaseolus vulgaris*) extruded mix diet; PEM, pea (*Pisum sativum*) extruded mix diet. Diets CAS-OB, KEM-OB, and PEM-OB were the same diets described above with added 5.48 g/day condensed milk. ^2^ E, effect of inclusion of extruded legume/rice/carob tree bean; O, effect of the obesogenic diet; E × O, interaction of both effects.

**Table 5:** Bile acids concentrations (μmol/g) in the feces of rats fed casein or casein plus extruded mix diets either obesogenic or not.

	Diet ^1^							*p-*Values ^2^		
	CAS	CAS-OB	KEM	KEM-OB	PEM	PEM-OB	Pooled SD	E	O	E × O
Cholic acid	0.088	0.134	0.072	0.082	0.093	0.055	0.058	0.021	0.414	0.043
Deoxycholic acid	0.112	0.124	0.784	0.805	0.946	0.614	0.208	<0.001	0.168	0.114
Chenodeoxycholic acid	0.005	0.007	0.003	0.003	0.003	0.002	0.004	0.002	0.391	0.177
Ursodeoxycholic acid	0.004	0.005	0.001	0.003	0.003	0.003	0.002	<0.001	0.279	0.800
Total	0.206	0.245	0.892	0.837	1.062	0.673	0.244	<0.001	0.129	0.041

Values are means (*n* = 12). ^1^ CAS, casein diet; KEM, kidney bean (*Phaseolus vulgaris*) extruded mix diet; PEM, pea (*Pisum sativum*) extruded mix diet. Diets CAS-OB, KEM-OB and PEM-OB were the same diets described above with added 5.48 g/d condensed milk. ^2^ E, effect of inclusion of extruded legume/rice/carob tree bean; O, effect of the obesogenic diet: E × O, interaction of both effects.

**Table 6:** Cholesterol and triglyceride concentrations in liver (mg/g fresh weight) and plasma (mg/dL) of rats fed casein or casein plus extruded mix diets either obesogenic or not.

	Cholesterol			Triglycerides	
	Liver	Plasma		Liver	Plasma
		LDL	HDL		
CAS ^1^	2.016	16.77	32.13	6.573	125.08
CAS-OB	1.911	15.20	31.50	7.880	203.46
PEM	1.771	11.48	29.53	5.364	144.19
PEM-OB	1.465	9.17	36.41	5.449	207.42
KEM	1.684	9.46	38.84	5.027	78.13
KEM-OB	1.475	12.05	37.02	6.231	146.71
Pooled SD	0.297	6.44	9.65	1.853	56.86
*p-Values*					
E ^2^	<0.001	0.01	0.191	0.001	0.240
O	0.017	0.776	0.730	0.050	<0.001
E*O	0.308	0.626	0.566	0.382	0.811

Values are means (*n* = 12). ^1^ CAS, casein diet; KEM, kidney bean (*Phaseolus vulgaris*) extruded mix diet; PEM, pea (*Pisum sativum*) extruded mix diet. Diets CAS-OB, KEM-OB, and PEM-OB were the same diets described above with added 5.48 g/day condensed milk. ^2^ E, effect of inclusion of extruded legume/rice/carob tree bean; O, effect of the obesogenic diet; E × O, interaction of both effects.

**Table 7:** Effect of the diet on liver long chain fatty acid (LCFA) composition of rats fed control diets (CAS) or diets supplemented with two extruded mixes of legumes plus cereals, normocaloric or obesogenic.

	Diet ^1^							*p-*Values ^2^		
	CAS	CAS-OB	PEM	PEM-OB	KEM	KEM-OB	Pooled SD	E	O	E × O
C10, capric acid	0.430	0.410	0.568	0.448	0.638	0.596	0.222	0.021	0.405	0.614
C14, myrisic acid	3.210	2.228	3.052	2.816	2.752	2.012	0.209	0.929	0.180	0.639
C15:1, cis-10-pentadecenoic acid	1.820	2.906	4.889	3.672	4.164	5.238	1.873	<0.001	0.289	0.182
C16, palmitic acid	17.194	19.919	23.474	21.570	21.595	23.237	3.586	<0.001	0.147	0.091
C16:1, palmitoleic acid	1.282	2.863	2.216	2.776	2.093	3.014	1.053	0.096	<0.001	0.121
C17, heptadecanoic acid	0.234	0.216	0.260	0.240	0.212	0.262	0.050	0.179	0.882	0.270
C17:1, cis-10-heptadecenoic acid	0.103	0.113	0.169	0.173	0.198	0.124	0.108	0.050	0.748	0.514
C18, stearic acid	11.132	10.027	16.108	13.345	15.229	14.576	2.989	<0.001	0.024	0.599
C18:1 9t, elaidic acid	0.203	0.196	0.214	0.211	0.188	0.289	0.068	0.171	0.287	0.143
C18:1 9c, oleic acid	11.702	15.513	12.890	13.486	12.866	15.230	3.164	0.991	0.003	0.167
C18:1 11c, ?	3.026	3.752	3.687	3.628	3.633	4.571	0.899	0.050	0.025	0.534
C18:2 n6 9c12c, linoleic acid	13.683	11.581	16.293	11.952	15.385	12.727	2.697	0.015	<0.001	0.224
C20, arachidic acid	0.048	0.040	0.061	0.043	0.055	0.046	0.012	0.025	<0.001	0.291
C18:3 n6, γ-linolenic acid	0.283	0.213	0.336	0.197	0.322	0.250	0.099	0.268	0.001	0.453
C20:1 n9, cis-11-eicosenoic acid	0.193	0.179	0.196	0.121	0.159	0.160	0.060	0.098	0.113	0.453
C20:2 n6, cis-11,14-eicosadienoic acid	0.446	0.284	0.471	0.293	0.455	0.313	0.123	0.505	<0.001	0.985
C22, behenic acid	0.032	0.032	0.038	0.030	0.035	0.039	0.013	0.391	0.747	0.796
C20:3 n6, cis-8,11,14-eicosatrienoic acid	0.375	0.311	0.554	0.427	0.4865	0.395	0.167	0.007	0.053	0.613
C20:4, arachidonic acid	4.907	2.610	0.011	0.022	0.003	0.093	3.589	<0.001	0.207	0.189
C24, lignoceric acid	0.044	0.040	0.046	0.034	0.044	0.062	0.047	0.252	0.041	0.169
C24:1 n9, nervonic acid	0.043	0.035	0.035	0.028	0.045	0.033	0.020	0.540	0.001	0.280
C22:6 n3, cis-4,7,10,13,16,19-docosahexaenoic acid	0.401	0.554	0.198	0.177	0.295	0.357	0.618	<0.001	0.185	0.223
SFA ^3^	54.588	50.131	51.849	56.261	51.156	48.422	5.114	0.875	0.198	0.068
MUFA	17.792	23.224	23.967	23.679	23.137	28.523	5.873	0.008	0.008	0.370
PUFA	20.380	16.009	19.876	14.839	18.074	15.515	3.950	0.268	<0.001	0.808

Values are means (*n* = 12). ^1^ CAS, casein diet; KEM, kidney bean (*Phaseolus vulgaris*) extruded mix diet; PEM, pea (*Pisum sativum*) extruded mix diet. Diets CAS-OB, KEM-OB, and PEM-OB were the same diets described above with added 5.48 g/day condensed milk. ^2^ E, effect of inclusion of extruded mix; O, effect of the obesogenic diet; E × O, interaction of both effects. ^3^ SFA, saturated fatty acids; MUFA, monounsaturated fatty acids; PUFA, polyunsaturated fatty acids.

**Table 8:** Effect of the diet on visceral fat long chain fatty acid (LCFA) composition of rats fed control diets (CAS) or diets supplemented with two extruded mixes of legumes plus cereals, normocaloric or obesogenic.

	Diet ^1^							*p-*Values ^2^		
	CAS	CAS-OB	PEM	PEM-OB	KEM	KEM-OB	Pooled SD	E	O	E × O
C10, capric acid	0.071	0.200	0.051	0.195	0.082	0.179	0.073	0.410	<0.001	0.679
C12, lauric acid	0.217	0.556	0.209	0.478	0.275	0.478	0.189	0.469	<0.001	0.167
C14, myristic acid	1.574	2.382	1.442	2.271	1.878	2.155	0.877	0.848	0.005	0.575
C14:1, myristoleic acid	0.173	0.203	0.134	0.230	0.164	0.179	0.100	0.676	0.162	0.700
C15, pentadecanoic acid	0.046	0.146	0.182	0.175	0.136	0.108	0.146	0.195	0.344	0.127
C16, palmitic acid	20.513	25.447	23.092	25.948	23.163	26.239	2.844	0.005	<0.001	0.090
C16:1, palmitoleic acid	4.812	6.281	5.164	7.011	5.541	6.305	1.105	0.074	<0.001	0.683
C17, elaidic acid	0.108	0.168	0.101	0.157	0.101	0.148	0.045	0.255	<0.001	0.654
C17:1, cis-10-heptadecenoic acid	0.034	0.012	0.015	0.012	0.009	0.013	0.018	0.068	0.077	0.068
C18, stearic acid	3.341	3.109	3.528	3.400	3.181	3.316	0.509	0.401	0.449	0.453
C18:1 9c, oleic acid	31.051	31.865	32.531	32.163	31.158	32.765	1.348	0.050	0.045	0.819
C18:1 11c, ?	1.737	2.651	1.952	2.528	1.778	2.277	0.687	0.696	<0.001	0.255
C18:2 n6, linoleic acid	33.343	23.807	28.625	22.592	29.967	23.246	4.962	0.006	<0.001	0.066
C18:3 n6, γ-linolenic acid.	0.115	0.082	0.173	0.126	0.081	0.095	0.073	0.461	0.307	0.786
C20:1, cis-11-eicosenoic acid	0.193	0.162	0.278	0.163	0.175	0.248	0.118	0.272	0.481	0.864
C18:3 n3, linolenic acid	0.417	0.344	0.361	0.341	0.534	0.469	0.233	0.455	0.403	0.778
C20:2 n6, cis-11,14-eicosadienoic acid	0.213	0.135	0.258	0.162	0.163	0.194	0.092	0.459	0.049	0.301
C20:4, arachidonic acid	0.671	0.423	0.565	0.411	0.375	0.212	0.290	0.082	0.027	0.695
SFA ^3^	26.104	31.269	28.896	32.876	29.025	32.838	3.414	0.002	<0.001	0.356
MUFA	37.991	41.281	40.091	42.185	38.876	41.823	1.974	0.004	<0.001	0.289
PUFA	34.602	24.588	29.634	23.374	30.939	24.104	5.197	0.006	<0.001	0.057

Values are means (*n* = 12). ^1^ CAS, casein diet; KEM, kidney bean (*Phaseolus vulgaris*) extruded mix diet; PEM, pea (*Pisum sativum*) extruded mix diet. Diets CAS-OB, KEM-OB, and PEM-OB were the same diets described above with added 5.48 g/day condensed milk. ^2^ E, effect of inclusion of extruded legume/rice/carob tree bean; O, effect of the obesogenic diet; E × O, interaction of both effects. ^3^ SFA, saturated fatty acids; MUFA, monounsaturated fatty acids; PUFA, polyunsaturated fatty acids.

## Abbreviations

CAS, casein diet; PEM and KEM, diets containing pea or kidney bean extruded mixes, respectively; CAS-OB, PEM-OB, and KEM-OB, obesogenic diets containing casein or pea or kidney bean extruded mixes, respectively.
